# Case Report: Robotic-Assisted Minimally Invasive Repair of a Direct Sliding Left Inguinal Hernia Containing Bladder and Colon

**DOI:** 10.7759/cureus.54975

**Published:** 2024-02-26

**Authors:** Anish M Varghese, Michael R Zemaitis, James Giannone, Marta B Sekh, Alex Barkan

**Affiliations:** 1 Medicine, St. George's University School of Medicine, St. George, GRD; 2 General Surgery, Richmond University Medical Center, Staten Island, USA; 3 Medicine, American University of Antigua, Osbourn, ATG

**Keywords:** direct sliding inguinal hernia, robot-assisted minimally invasive surgery (ramis), robotic assisted hernia repair, sliding inguinal hernia, inguinal hernias

## Abstract

A direct sliding inguinal hernia descends through the superficial inguinal ring and encroaches on nearby organ structures, such as the bladder. This type of hernia is rare with a 2-5% incidence and occurs due to a weakness within the lower abdominal wall, usually associated with advancing age, that permits the distal colon to descend into the inguinal canal. Direct sliding inguinal hernias are a rare subset of inguinal hernias that require meticulous dissection due to their incorporation of nearby organs such as the bladder or colon. Few cases report repair of these hernias laparoscopically; however, the use of a hybrid laparoscopic/open approach has not been extensively documented and it may be beneficial to explore the use of this approach in inguinal hernia repair.

We present a case of a robotic-assisted minimally invasive repair of a direct sliding inguinal hernia in an 85-year-old male. He initially presented to the emergency department with left-sided groin pain and imaging revealed he had a direct sliding inguinal hernia that incorporated the bladder wall. He was admitted to surgery for a robotic-assisted minimally invasive inguinal hernia repair with mesh. During the surgery, after seeing the extent at which the hernia sac incorporated the bladder wall, the procedure was converted to an open approach to perform the remainder of the reduction; however the robot was reintroduced for mesh placement. Post-operatively, the patient experienced mild incisional abdominal pain with return of bowel function on day four and was discharged that same day.

## Introduction

A sliding inguinal hernia is defined as a subset of inguinal hernias in which a portion of the hernia sac wall encloses an organ. This type of hernia occurs primarily due to a weakness or defect within the lower abdominal wall, usually associated with advancing age, that permits the distal descending or sigmoid colon to protrude through the lower abdominal wall and descend into the inguinal canal. However, unlike other inguinal hernias, a direct sliding inguinal hernia descends through the superficial inguinal ring and encroaches on nearby organ structures, such as the bladder. Unlike inguinal hernias, these hernias are extremely rare with a 2-5% incidence [1-3].

Untreated inguinal hernias have a high risk of incarceration or strangulation; a strangulated inguinal hernia can be life-threatening and therefore requires urgent surgical intervention. Direct sliding inguinal hernias present an additional challenge as their compression on nearby structures can present with increased urinary frequency, dysuria, or urinary retention. Additionally, direct bladder compression by the hernia may result in ureteral dilatation, hydronephrosis, and post-renal acute kidney injury [4-6]. Surgical approaches to repair a sliding inguinal hernia include the Lichtenstein tension-free mesh technique in which post-reduction a mesh is used to patch the abdominal wall defect rather than suturing the borders of the defect together [7]. Over time, the mesh acts as a scaffold in which new tissue grows over to reinforce the abdominal wall resulting in a strong repair that is less likely to require reconstruction. This technique has been adopted to be performed in open, laparoscopic, and robotic laparoscopic settings; however, we will focus our attention on the traditional and robotic laparoscopic approaches.

In the traditional laparoscopic approach, a few small 5-8 mm incisions are made on the lower abdomen and special surgical instruments are introduced into the abdominal cavity which allows the surgeon to visualize the abdominal cavity, sharply or bluntly dissect tissue, cauterize, and suture intra-abdominally. This approach was first described by Ger in 1991, however more recently in 2015, Dominguez et al. demonstrated robotic inguinal hernia repair as an alternative approach to traditional laparoscopy. This robotic approach appeared to demonstrate benefits for patient outcomes, such as decreased postoperative pain, and for surgeon ergonomics, such as reducing excessive movement to manipulate laparoscopic instruments [8,9]. Here, we present a case of a robotic-assisted minimally invasive repair of a direct sliding inguinal hernia containing a bladder. 

## Case presentation

**Figure 1:**
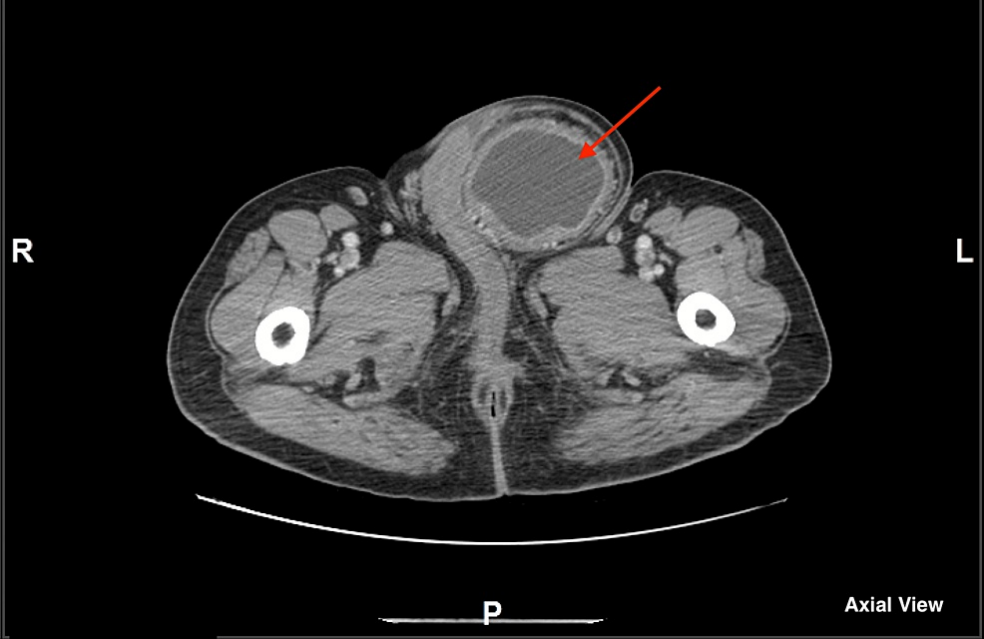
CT Abdomen and Pelvis with contrast (Axial View) Large left inguinal hernia containing a significant part of the bladder (red arrow).

The patient is an 85-year-old South Asian male with a past medical history of diabetes mellitus, hypertension, and benign prostatic hyperplasia and a past surgical history of a transurethral resection of the prostate in January 2022 who presented to the emergency department with left-sided groin pain. He reported that although he was aware that he had a hernia, only recently had it become painful. An abdominal/pelvic CT (Figure 1) was performed and it revealed a large left inguinal hernia that contained a significant part of the bladder medially. It also revealed that the neck of the hernia was 2.5 cm and that the bladder wall was markedly thickened to 1.7cm and measured 8.4 x 11 x 7.2 cm. A diagnosis of a direct sliding left inguinal hernia was made and the patient was admitted to surgery for robotic-assisted minimally invasive inguinal hernia repair with mesh. 

The patient was positioned supine, placed in 15-degree Trendelenburg on the operating table, shaved, and the surgical site was prepped with povidone-iodine and draped. Using the Hasson open technique, a 12 mm incision was made near the umbilicus and a blunt tip trocar was inserted into the abdomen to serve as a laparoscopic camera port. Three incisions, each 8 mm long, were made on the left and right mid abdomen. A fourth incision measuring 5 mm was made on the right mid abdomen. After inserting the robot laparoscopic ports, the DaVinci XI robot was then introduced into the field. Upon entry, the hernia was easily isolated, freed from the lateral wall and partially reduced. However, after about two hours of dissection, it became apparent that the medial wall of the hernia sac extensively incorporated the bladder. Thus, a decision was made to perform the remainder of the reduction via open procedure. A counter incision was made from the left anterior superior iliac spine (ASIS) to the pubic tubercle and the medial surface of the hernia was successfully freed from the bladder wall and the remainder of the hernia was reduced. Post-reduction, the robot was then re-introduced into the field and a 10 x 15 cm left-sided ProGrip self-gripping polyester mesh was placed over the hernia defect and the peritoneal flap was sutured via 2-0 absorbable V-lock sutures intra-abdominally. Insufflation pressure was then reduced to 5 mm Hg and hemostasis was confirmed prior to closure. 

The surgery had a total operative time of four hours and 56 minutes and an estimated blood loss of 50 cc. A large portion of the surgery was dedicated to meticulously separating the bladder wall from the medial aspect of the hernia sac. After two hours of attempting to dissect the medial wall of the hernia and being unable to completely reduce the hernia robotically, a decision was made to perform the remainder of the reduction under open procedure. Post-operatively, the patient was noted to have scrotal swelling which was expected. He also complained of mild incisional abdominal pain and distension. His abdominal X-ray was suggestive of ileus. On postoperative day number four, he had return of bowel function. At this time he was started on a clear liquid diet which was advanced as tolerated. He was then cleared by physical therapy for discharge the same day. 

## Discussion

Direct sliding inguinal hernias are an extremely rare occurrence and if untreated, can lead to devastating complications such as bowel strangulation, bowel necrosis, bowel perforation, sepsis, post-renal kidney injury, and kidney failure to name a few. When the hernia is compressing on the bladder and is left untreated, the transversalis fascia and external oblique aponeurosis encompassing the bowel becomes tightly adhered to the bladder wall. Therefore, during dissection, the medial wall of the hernia sac must be meticulously separated to prevent damage to the bladder and potential leakage of bladder contents into the peritoneal cavity. To aid in the dissection, surgeons may opt to perform the procedure laparoscopically to intrabdominally free the hernia from the inguinal canal wall and bladder. This approach allows the surgeon to create a tunnel through the fascia for which the descending colon and bladder can be reduced back into the abdomen [10]. In the described case, however, the bladder could only be partially reduced robotically; once we saw the extent to which the hernia sac was adhered to the bladder wall, a decision was made to perform the remainder of the reduction under open procedure. 

Although this decision was made intraoperatively, there are several cases where a planned hybrid laparoscopic approach was implemented for giant incisional ventral hernia repair. Zachariah et al. and Yoshikawa et al. reported minimal postoperative complications from a hybrid technique especially in patients with comorbidities such as systemic hypertension, type 2 diabetes mellitus, chronic liver disease with portal hypertension, dyslipidemia, and chronic obstructive pulmonary disease. However, Zachariah et al. aimed to propose the use of a single-incision laparoscopic hybrid approach in which a single periumbilical incision and raising flaps allowed them to introduce and properly position all their ports [11]. Yoshikawa et al. demonstrated a hybrid approach for ventral hernia repair in which intestinal adhesions were removed laparoscopically but then monofilament thread was subcutaneously introduced into the abdominal cavity for hernia defect closure instead of through the laparoscopic ports. They reported this approach being feasible for defects larger than 15 cm and in patients with a BMI >25 kg/m^2 [12]. Yane et al. described a case in which the incarcerated small intestine was reduced laparoscopically but mesh repair was performed via an anterior open approach because they found the right inferior epigastric vein was severely varicotic and it was hazardous to detach the anterior peritoneal cavity intraabdominally. Via this approach, they reported no intra- or postoperative complications [13]. Complications may arise due to variability in course and anastomosis of inferior epigastric vessels. Usually, the inferior epigastric artery arises independently from the external iliac artery in 83.6%; it may also arise dependently from the common trunk of external iliac artery with the obturator artery or aberrant obturator artery in 15.1.8 % or 1.3%. Furthermore, the inferior epigastric artery was found to have obturator and aberrant obturator branch in 3.3 % and 0.3 % of the cases. Therefore, the arterial connection of the inferior epigastric artery and obturator or its accessory branch is 20% [14]. As the retropubic area contains great vascular variability, a great precaution is required before pubic surgical procedures such as internal fixation of pubic fracture, an inguinal hernia repair.

## Conclusions

We present a case of a direct sliding inguinal hernia containing the bladder in an elderly South Asian male with a past medical history of benign prostatic hyperplasia, hypertension, and diabetes mellitus and a recent surgical history of transurethral resection of the prostate. The initial plan was to free the hernia sac laterally and medially and to perform the reduction robotically laparoscopically. However, once we visualized the extent that the medial wall incorporated the bladder, a decision was made to convert to open procedure to free the hernia sac and perform the remainder of the reduction. The robot was then reintroduced into the field so the mesh can be inserted and positioned laparoscopically. This allowed us to meticulously position the mesh over the borders of the hernia defect and to ensure adequate coverage prior to suturing the mesh in place. Although this decision was made intraoperatively based on circumstance, given the minimal postoperative complications in this patient as well as the reported advantages of a planned hybrid laparoscopic approach in ventral hernia repair, it may be beneficial to explore the use of a planned hybrid laparoscopic approach in inguinal hernia repair. However, future studies would need to implement this particular approach on a larger scale and quantify the advantages, disadvantages, and postoperative complications in chronically incarcerated inguinal hernia repair. 
